# Quality of DNA Extracted from Mouthwashes

**DOI:** 10.1371/journal.pone.0006165

**Published:** 2009-07-07

**Authors:** Tetyana Zayats, Terri L. Young, David A. Mackey, François Malecaze, Patrick Calvas, Jeremy A. Guggenheim

**Affiliations:** 1 School of Optometry and Vision Sciences, Cardiff University, Wales, United Kingdom; 2 Duke University, Center for Human Genetics, Durham, North Carolina, United States of America; 3 Centre for Eye Research Australia, University of Melbourne, Royal Victorian Eye and Ear Hospital, Melbourne, Australia; 4 Department of Ophthalmology, University of Tasmania, Royal Hobart Hospital, Hobart, Australia; 5 Inserm U563 Hopital Purpan CHU Toulouse, Toulouse, France; Leiden University Medical Center, Netherlands

## Abstract

**Background:**

A cost effective, safe and efficient method of obtaining DNA samples is essential in large scale genetic analyses. Buccal cells are an attractive source of DNA, as their collection is non-invasive and can be carried out by mail. However, little attention has been given to the quality of DNA extracted from mouthwashes.

**Methodology:**

Mouthwash-derived DNA was extracted from 500 subjects participating in a genetic study of high myopia. DNA quality was investigated using two standard techniques: agarose gel electrophoresis and quantitative polymerase chain reaction (qPCR).

**Principal Findings:**

Whereas the majority of mouthwash-derived DNA samples showed a single band of high molecular weight DNA by gel electrophoresis, 8.9% (95% CI: 7.1–10.7%) of samples contained only a smear of low-to-medium molecular weight, degraded DNA. The odds of DNA degradation in a subject's second mouthwash sample, given degradation of the first, was significantly greater than one (OR = 3.13; 95% CI: 1.22–7.39; Fisher's test P = 0.009), suggesting that DNA degradation was at least partially a subject-specific phenomenon. Approximately 12.4% (95% CI: 10.4–14.4%) of mouthwash-derived DNA failed to PCR amplify efficiently (using an ∼200 bp microsatellite marker). However, we found there was no significant difference in amplification success rate between DNA samples judged to be degraded or non-degraded by gel electrophoresis (Fisher's test P = 0.5).

**Conclusions:**

This study demonstrated that DNA degradation affects a significant minority of saline mouthwashes, and that the phenomenon is partially subject-specific. Whilst the level of degradation did not significantly prevent successful amplification of short PCR fragments, previous studies suggest that such DNA degradation would compromise more demanding applications.

## Introduction

In large-scale genetic linkage and association studies there is a need for a cost-effective, safe and efficient method of obtaining DNA. An attractive approach is to use buccal cells as, in comparison to blood, they offer a non-invasive and more easily collected source of cellular material. Various methods of buccal cell collection have been proposed, such as mouthwash, cytobrush and type cards [1], [2]. Among these procedures mouthwash can be performed by study participants without supervision, has the advantage of being collected via mail [3], [4] and yields the highest amount of DNA [1].

Despite these numerous advantages, there is a need for caution in using DNA extracted from buccal cells. The presence of non-human DNA in mouthwash samples, e.g. from oral bacteria or food remnants, has been shown to lead to the overestimation of human DNA yield [5]. Thus, quantitative PCR (qPCR) with human-specific primers has proved to be a useful guide for determining human DNA quantity prior to high-throughput analysis. Effective PCR amplification also suggests good DNA quality. However, the use of buccal DNA for more demanding, large-scale genetic applications can be problematic [5], [6]: for instance, DNA that is of poor quality due to degradation can lead to incorrect or missing genotype calls, discordant results in samples subjected to whole genome amplification, and difficulties in using the samples in DNA-pooling experiments [7], [8].

Together, the above results suggest that the quality of mouthwash-derived DNA is inferior to that obtained from blood. However, apart from studies that have assessed PCR efficiency, this issue has received little attention. Feigelsen and co-workers [5] reported studying DNA degradation in a sample of 24 subjects using gel electrophoresis, but did not include any results of this evaluation in their paper. This study sought to examine the issue of buccal DNA quality in greater detail by carefully assessing the amount of DNA degradation in samples collected as part of an established molecular genetic study [9], [10].

## Materials and Methods

### Subjects

Ethical approval for the study was granted by the Cardiff University Human Sciences Research Ethics Committee. The study adhered to the tenets of the Declaration of Helsinki, and all participants provided written informed consent.

Each of 500 participants in the UK arm of an international, multi-centre genetic study of high myopia [11] provided two mouthwashes (see below). Subjects were asked to mail their samples back to the laboratory as soon as possible, and the samples were processed on the day of arrival.

### Mouthwash procedure and DNA extraction

Subjects were supplied with two 50 ml skirted tubes each containing 15–20 ml of sterile 0.9% NaCl. For each tube in turn, subjects were instructed to swish the saline vigorously in the mouth for 20–30 seconds, before spitting it back into the same tube. To maximize DNA yield, participants were requested to perform the mouthwash rinses first thing in the morning before brushing their teeth, eating or drinking [5]. On arrival at the laboratory, mouthwash samples were refrigerated at 4°C or at least 40 minutes, and then centrifuged at 3500 rpm for 5 minutes in a Boeco C-28 centrifuge (Boeckel & Co, Hamburg, Germany). The supernatant was discarded, and the buccal cell pellet resuspended in 480 µl of Extraction Buffer (10 mM tris-HCl, pH 8.0, 1 mM EDTA, 0.5% SDS) and frozen at −20°C until processed further. Upon thawing, 20 µl of proteinase-K (10 mg/ml) was added to each cell suspension and incubated in a waterbath with continuous shaking (∼100 rpm) at 37°C for 2 hours. To separate insoluble material, tubes were centrifuged at 14000 rpm for 3 minutes and the supernatant was transferred to a fresh Eppendorf tube containing ∼25 µl high vacuum grease (Dow Corning Ltd). The vacuum grease served as a barrier between the aqueous and organic phases during phenol/chloroform extraction, which was performed and repeated until the supernatant was clear. After the addition of 19 µl 5 M NaCl and 1 ml 100% ethanol, the DNA was precipitated overnight at −20°C and then centrifuged at 14000 rpm for 10 minutes. The supernatant was removed and the DNA pellet was washed with 1 ml ice-cold 70% ethanol. After air-drying for 3 minutes, the DNA pellet was dissolved in 100 µl of TE (10 mM tris, 1 mM EDTA, pH 8.0) and incubated at 37°C for 30 minutes with periodic gentle mixing.

### Agarose gel electrophoresis

A 1 µl aliquot of each mouthwash-extracted DNA sample was diluted 1∶10 in TE. Ten microlitres of the dilution was then mixed with 2.4 µl of Loading Buffer (15% Ficoll 400, 0.5% xylene cyanol FF, 10 mM EDTA, containing a 1∶50 dilution of stock SYBR Green I (Molecular Probes-Invitrogen Ltd, Paisley, UK)) and 10 µl of the mixture was electrophoresed on a 1% agarose gel at 60 Volts for 30 minutes. A DOC-008.XD (UVItec Ltd, Cambridge, UK) camera system coupled to an ultraviolet transilluminator was used to take a digital photograph of the gel and degradation was then scored by visual inspection (performed twice by two observers independently of one another: the results were fully concordant).

### Quantitative Polymerase Chain Reaction (qPCR)

A measure of the human DNA content of mouthwash-derived DNA samples was obtained in one of two ways: a standard qPCR reaction followed by agarose gel and scanning densitometry, or a real-time qPCR reaction.

For the standard reaction, samples were diluted with a known volume of TE to give an expected final DNA concentration in the range 1–5 ng/µl, and 7.5 µl of this template DNA solution was mixed with 7.5 µl PCR mastermix to give final concentrations: 1× HotStar PCR buffer (Qiagen Ltd, Crawley, UK), 1.5 mM MgCl2, 200 µM each dNTP, 1 µM of each primer. Each reaction contained 0.5 U HotStar Taq polymerase. Amplification was achieved using 25–27 cycles of PCR (denaturation at 94°C for 1 minute, annealing at 60°C for 1 minute and extension at 72°C for 1 minute) after a preliminary step of 15 minutes at 95°C to activate the enzyme. The following primers were used to amplify a human-specific amplifier corresponding to a microsatellite marker on chromosome 7 (D7S3056): forward 5′ CAA TAG CCC TGA CCT TAT GC, reverse 5′ TAC CTA CCT ACC TAC CTC TAT GGC. PCR products were mixed with 3.75 µl Loading Buffer (as above) and separated on a 2% agarose gel. Human genomic DNA standards (0, 2, 4 or 6 ng template DNA) were included on each qPCR plate and gel to allow samples to be quantified by gel densitometry (QuantityOne software, GE Healthcare UK Ltd, Chalfont St. Giles, UK).

For real-time qPCR, amplification was carried out using a Rotor-Gene 6000 thermal cycler, with SYBR-Green I as the fluorophore. Quantification of DNA was achieved by constructing a standard curve of calculated Ct versus concentration for a set of DNA standards that were included in each run. Reaction reagents were mixed to give final concentrations: 1.2× HotStar PCR buffer, 3 mM MgCl2, 0.24 mM dNTPs mix, 1.2 µM of each primer (D7S3056) and 1∶40 000 SYBR Green I. Each 10 µl reaction contained 1 U HotStar Taq polymerase and mouthwash-extracted DNA diluted with a known volume of TE to an expected concentration of 0.5–2.5 ng/µl. Amplification was achieved using 40 cycles of PCR (denaturation at 94°C for 1 minute, annealing at 60°C for 1 minute and extension at 72°C for 1 minute) after a preliminary step of 10 minutes at 95°C to activate the enzyme.

### High-throughput SNP Array Genotyping

Mouthwash DNA from 253 participants that were judged as non-degraded by gel electrophoresis was sent to the Centre for Inherited Disease Research (CIDR) for genotyping on the Illumina 6 k Human Bead array [12]. Details of the genotyping procedures are available at http:/www.cidr.jhmi.edu/human_snp.html. The proportion of mouthwash-derived DNA samples that were successfully genotyped was compared to the results of blood-derived DNA sent at the same time. Genotyping was deemed successful if the sample passed the quality control assessment carried out by CIDR. This was based on the use of Illumina's BeadStudio software GenCall (GC) score (a GC score ranges from 0 to 1 and reflects the proximity within a cluster plot of intensities of that genotype to the centroid of the nearest cluster). All genotypes with GC score below 0.25 were considered as failures. DNA samples with >4% genotyping failures were judged as failed samples.

### Statistical Analysis

Since DNA degradation appeared as an all-or-nothing event as judged from agarose gels, mouthwashes were scored as either intact or degraded using a binary code. Fisher's exact test and odds ratio were calculated for a 2×2 table containing counts of the number of first and second degraded DNA samples from the 2 consecutive mouthwashes provided by each subject.

The results of qPCR were analyzed in the same way: the binary coding for qPCR reactions was based on whether or not mouthwash-extracted DNA samples achieved a threshold level of amplification (the threshold level having been chosen to approximate the minimum amplification level sufficient for successful microsatellite genotyping). Fisher's exact test and odds ratio were computed for a 2×2 table comprising the number of successful qPCR reactions when template DNA was or was not degraded.

## Results

**Figure 1:**
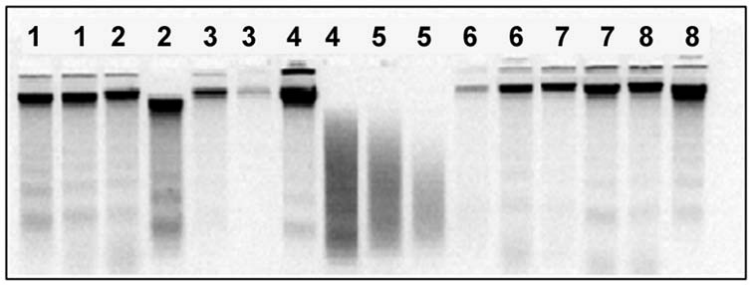
Gel electrophoresis of mouthwash-extracted DNA. Agarose gel electrophoresis of DNA extracted from mouthwashes of 8 subjects (2 samples per subject). Subjects are identified by the figures above lanes. DNA extracted from one of the mouthwashes provided by subject 4 and both mouthwashes provided by subject 5 was found to be degraded.

DNA was successfully extracted from all mouthwashes and analyzed by gel electrophoresis. Degradation was observed in a proportion of samples, evident as a broad smear of fluorescence in place of the usual single, sharp, high molecular weight band (Figure 1). The frequency of DNA sample degradation was 8.9% (95% CI: 7.1–10.7%; N = 1000). Specifically, 52 of the 500 first mouthwashes (10.4%) and 37 of the 500 second mouthwashes (7.4%) were degraded (Table 1). The odds ratio for DNA degradation in the second sample given degradation of the first sample was 3.13 (95% CI: 1.22–7.39), which was statistically significant (P = 0.009, Fisher's exact test).

**Table 1:** DNA degradation in a subject's first mouthwash sample when analyzed as a risk-factor for DNA degradation in their second mouthwash sample.

	DNA degraded in 2^nd^ mouthwash	DNA non-degraded in 2^nd^ mouthwash	Total
DNA degraded in 1^st^ mouthwash	9	43	52
DNA non-degraded in 1^st^ mouthwash	28	420	448
Total	37	463	500

**Figure 2:**
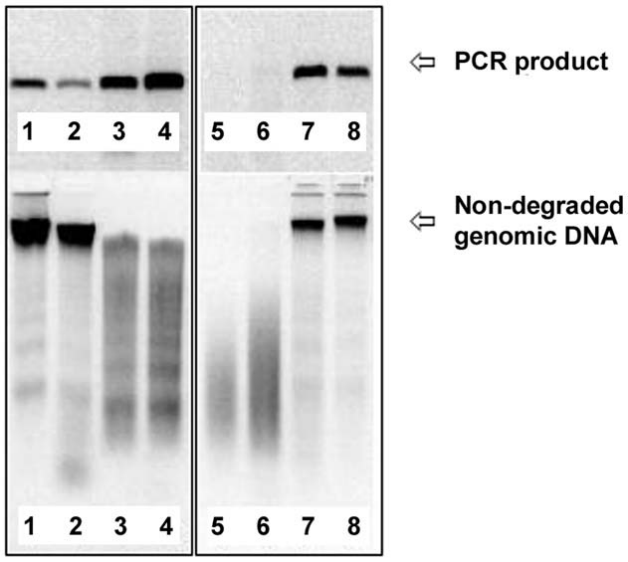
PCR efficiency of degraded DNA samples. Eight mouthwash DNA samples (lower panels) were used as templates for PCR amplification (upper panels). Partially degraded DNA samples containing residual high molecular weight DNA typically permitted efficient PCR amplification (lanes 3 and 4). Severely degraded DNA typically failed to PCR amplify (lanes 5 and 6).

Each DNA sample was also assessed using a qPCR assay (with primers targeting a human microsatellite marker). Samples were scored as having “passed” or “failed” to amplify efficiently, depending on whether they reached a threshold level (this threshold being chosen as representative of the minimum level of PCR product required for successful microsatellite genotyping). For the 1000 mouthwash-derived DNA samples tested in total, 85.4% of degraded samples passed the qPCR test, compared with 87.8% of non-degraded samples. Statistical analysis suggested that PCR amplification of degraded samples did not differ significantly from that of non-degraded ones (P = 0.5; Fisher's exact test; Table 2). The presence of at least some high molecular weight DNA by gel electrophoresis was associated with successful qPCR amplification (Figure 2), although this was not investigated in detail.

**Table 2:** PCR success in degraded and non-degraded DNA samples.

	Successful PCR	Failed PCR	Total
Degraded DNA	76	13	89
Non-degraded DNA	800	111	911
Total	876	124	1000

Of 253 mouthwash DNA samples that were genotyped using the Illumina 6 k SNP array platform, all except one sample (99.6%) were genotyped successfully (i.e. at least 96% genotypes called). For DNA extracted from blood and sent for genotyping at the same time, between 0.6–5.3% DNA samples could not be genotyped, depending on the collection center. For the 252 mouthwash DNA samples that were successfully genotyped, the average number of SNPs that could be genotyped for each subject was 99.7%, and the reliability of SNP genotyping “blind” duplicate mouthwash DNA samples was high (>99.9% concordance).

## Discussion

A major finding from this study was the discovery that ∼10% of DNA samples obtained from saline mouthwashes contained degraded DNA. Furthermore, there was an approximately 3-fold increased risk of DNA degradation in a subject's second mouthwash sample, given DNA degradation in their first. This finding suggests that DNA degradation may be due to one or more factors specific to individual subjects, implying that DNA may always be degraded in the mouthwash samples of such participants. If such DNA degradation is not detected prior to certain downstream analyses, it is likely to lead to a failure rate of ∼10% of samples. For high-throughput SNP genotyping and whole genome amplification reactions, this will result in reduced statistical power compared to that anticipated. For DNA pooling experiments it may lead to suboptimal results, since fewer individuals will contribute to the genotyping signals than expected.

Variability in the quality of DNA obtained from mouthwashes could arise due to dissimilarity in each individual's oral flora, dietary or lifestyle habits, differences in desquamation of oral mucosa [6] or because of other reasons, such as how exactly the mouthwash rinsing protocol was performed, the composition of the mouthwash solution, and the lag time between mouthwash rinsing and processing. There is a highly diverse and subject-specific, bacterial flora in the healthy oral cavity [13], [14] that can be affected by smoking [15], [16] and diet [17], and which in turn can lead to DNA damage [18].

The way in which the mouthwash rinsing procedure is performed has been shown to significantly affect DNA yield [19], [20]. Furthermore, cells recovered in mouthwashes are likely to be superficial ones in the process of apoptosis: about 30% of buccal cells collected from persons with healthy, non-inflammatory oral mucosa show apoptotic signs [21]. Therefore, DNA from certain individuals may be more prone to the signs of DNA degradation noted here.

Lag time between mouthwash rinsing and processing has been proposed as a possible cause of poor DNA quality [1], [5], [22]. However, storage of unprocessed mouthwashes at room temperature for up to 1 week has been shown not to affect DNA yield or the efficiency with which the DNA can be amplified by PCR [5], [22]. Resistance of DNA to degradation over time is presumably influenced by the composition of the mouthwash solution itself (e.g. the presence or absence of alcohol). Nonetheless, DNA is stable in saline at room temperature for up to 4 days [20]. Finally, tests carried out in our laboratory on a small group of volunteer subjects, each of whom provided one mouthwash sample per day over a period of 12 days, and whose mouthwashes were extracted after 0, 1, 2 or 3 days of storage at room temperature, were consistent with DNA degradation being subject-specific, but unrelated to lag time prior to DNA extraction (data not shown).

Approximately 12% of the DNA samples examined in this study failed to amplify with qPCR. Interestingly, this failure appeared to be independent of visible DNA degradation, suggesting that factors other than this are to blame [23]. It is likely, that carry-over of contaminating substances from DNA extraction played a major role in failure of qPCR in our mouthwash samples. During purification with phenol-chloroform, poor PCR performance [24] and relative loss of human DNA [19] have been observed. In our experience, re-extraction improved PCR performance in approximately 50% of cases, supporting the possible presence of carry-over inhibitors in a minority of DNA samples.

### Conclusion

We found that approximately 10% of mouthwash samples collected using a standardized protocol in our laboratory exhibited signs of DNA degradation. The phenomenon was shown to be partially subject-specific, although further work will be required to trace the precise cause(s) involved. For samples of mouthwash-derived DNA that did not show signs of DNA degradation by gel electrophoresis and that amplified efficiently using our human-specific qPCR assay, high throughput genotyping results were comparable to those obtained for DNA extracted from blood. Thus, we conclude that DNA quality is not uniform amongst mouthwash samples: most mouthwash-derived DNA samples contain high quality DNA, but others contain DNA that is heavily degraded. We suggest that screening for DNA degradation be undertaken prior to the use of mouthwash-derived DNA for demanding applications such as high-density SNP genotyping, whole genome amplification, and DNA pooling experiments.
